# Use of Grafted Voltage Stabilizer to Enhance Dielectric Strength of Cross-Linked Polyethylene

**DOI:** 10.3390/polym11010176

**Published:** 2019-01-20

**Authors:** Wei Dong, Xuan Wang, Bo Tian, Yuguang Liu, Zaixing Jiang, Zhigang Li, Wei Zhou

**Affiliations:** 1 Key laboratory of Engineering Dielectrics and Its Application, Ministry of Education, College of Electrical and Electronic Engineering, Harbin University of Science and Technology, Harbin 150080, China; dongweifree@163.com; 2 Technical Physics Institute of Heilongjiang Academy of Sciences, Harbin 150086, China; tianbo_0627@163.com (B.T.); ygliu_63@163.com (Y.L.); lzg0015@gmail.com (Z.L.); zhouwei5605@163.com (W.Z.); 3 Department of Polymer Science and Technology, School of Chemical Engineering and Technology, Harbin Institute of Technology, Harbin 150001, China

**Keywords:** cross-linked polyethylene, grafted voltage stabilizer, breakdown strength, electrical tree initiation voltage

## Abstract

Aromatic voltage stabilizers can improve the dielectric properties of cross-linked polyethylene (XLPE); however, their poor compatibility with XLPE hinders their practical application. Improving the compatibility of aromatic voltage stabilizers with XLPE has, therefore, become a new research goal. Herein 1-(4-vinyloxy)phenylethenone (VPE) was prepared and characterized. It can be grafted onto polyethylene molecules during the cross-linking processes to promote stability of the aromatic voltage stabilizers in XLPE. Fourier transform infrared spectroscopy confirmed that VPE was successfully grafted onto XLPE, and effectively inhibited thermal migration. Thermogravimetric analysis showed that the grafted VPE/XLPE composite exhibits a better thermal stability than a VPE/PE blend composite. Evaluation of the electrical properties showed that the breakdown strength and electrical tree initiation voltage of the VPE/XLPE composite were increased by 15.5% and 39.6%, respectively, when compared to those of bare XLPE. After thermal aging, the breakdown strength and electrical tree initiation voltage of the VPE/XLPE composite were increased by 9.4% and 25.8%, respectively, in comparison to those of bare XLPE, which indicates that the grafted voltage stabilizer can effectively inhibit its migration and enhance the stability of the composite material.

## 1. Introduction

Cross-linked polyethylene (XLPE) is widely applied as a cable insulation material due to its superior insulating properties, electrical properties, mechanical properties and cost effectiveness. However, electrical tree will emerge during operation as an important electrical breakdown mechanism of the insulation material [1,2,3,4,5]. It propagates through XLPE in the form of branches, and this process can result in the failure of the insulation material, which heavily influences the operating security and lifetime of the cables [6,7,8,9,10].

Electrical tree is induced by voids and impurities in the insulation material. Some of these problems have been addressed by making the insulation material very clean and minimizing the voids and impurities [11]; however, it is very difficult to further improve the dielectric properties of the insulation material by this method. Inorganic nano-fillers have been widely reported to especially improve the breakdown strength and electrical tree initiation voltage of insulation materials [12,13,14,15]; agglomeration and cavitation in polymer nanocomposites can be prevented by controlling the interface chemistry. A study by Liu [16,17] found that aluminum oxide nanoparticles coated with alkyl-terminated silanes having alkyl groups can be uniformly dispersed in a polyethylene matrix, resulting in nanocomposites with improved dielectric properties.

Suitable additives that can inhibit electrical tree, termed as voltage stabilizers [18,19,20,21], have been widely studied. Adding voltage stabilizers is the most feasible way to improve the dielectric properties of insulation materials. Jarvid [22,23] states that high electron affinity is the design criterion for voltage stabilizers; fullerenes have high electron affinity, which makes them potential voltage stabilizers. Jarvid’s studies have shown that fullerenes are very efficient voltage stabilizers and can find potential applications in power cables. Most voltage stabilizers have an aromatic or benzophenone-like structure, such as acetophenone (AP) [19,24] or benzyls [25], which can effectively inhibit the formation of electrical tree; however, these voltage stabilizers have poor compatibility with XLPE, which causes them to migrate out of XLPE, influencing the lifetime and properties of the insulation material. The issue of voltage stabilizer migration has recently attracted considerable attention. Jarvid et al. [26,27] synthesized a benzil-type compound with a larger alkoxy chain as a voltage stabilizer in XLPE. This compound effectively inhibited electrical tree initiation and showed improved compatibility with XLPE; however, it did not inhibit the migration of voltage stabilizers. Zhang et al. found by theoretical calculation that acetophenone doping in XLPE or grafting onto XLPE molecule chains can suppress electrical tree initiation and propagation and enhance the breakdown strength [28,29]. We propose to address the problem of voltage stabilizer migration by grafting the voltage stabilizer onto XLPE, which to our knowledge has not been previously reported.

The irradiation crosslinking reaction of polyethylene was completed at room temperature without adding a crosslinking agent. The advantages of irradiation cross-linking compared with traditional methods include energy saving without waste production and a fast processing speed. In this study, we modified 4′-hydroxyacetophenone (HAP) by introducing a side chain containing an active double-bond group in the para position, allowing it to be grafted onto the polyethylene (PE) molecule chain during the radiation cross-linking process, thus yielding 1-(4-vinyloxy)phenylethenone (VPE). This yielded a VPE/XLPE composite with significantly improved breakdown strength and electrical tree initiation voltage, and suppressed migration of the voltage stabilizer.

## 2. Materials and Methods

### 2.1. Materials

LDPE was purchased from China petrochemical group co., Ltd. (Beijing, China). 4′-hydroxyacetophenone was purchased from Aladdin industrial corporation. (Shanghai, China). 1,2-dichloroethane was purchased from Tianjin fuyu fine chemical co., Ltd. (Tianjing, China). Benzyltriethylammonium chloride (TEBAC) was purchased from Aladdin industrial corporation (Shanghai, China). NaOH was purchased from Tianjin Guangfu technology development co., Ltd. (Tianjin, China). petroleum ether, was purchased from Tianjin fuyu fine chemical co., Ltd. (Tianjing, China). ethyl acetate was purchased from Aladdin industrial corporation (Shanghai, China). silica gel was purchase from Qingdao ocean chemical Co., Ltd. (Qingdao, Shandong, China).

### 2.2. Preparation of VPE

**Scheme 1:**
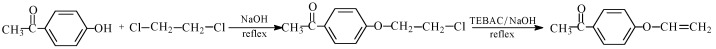
Synthesis of VPE.

VPE was prepared as illustrated in Scheme 1. 4′-hydroxyacetophenone was dissolved in 1,2-dichloroethane. 50% NaOH aqueous solution was then added. The mixture was continuously stirred for 5 h at 83.5 °C. The dark red 1-(4-(2-chloroethoxy)phenyl)ethanone was collected by distillation. The product was washed with distilled water several times, and dried at 80 °C in a vacuum for 6 h. 1-(4-(2-chloroethoxy)phenyl)ethanone and TEBAC were added into 50% NaOH aqueous solution; the mixture was stirred at 100 °C for 6 h, and the product was separated through column chromatography isolation to obtain VPE, the stationary phase is 40 mesh column grade silica gel and the mobile phase is 10:1 petroleum ether and ethyl acetate mixed solution.

### 2.3. Preparation of VPE/XLPE

**Scheme 2:**
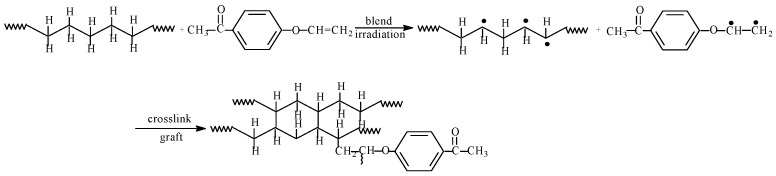
VPE grafted onto XLPE.

APE/XLPE was prepared as illustrated in Scheme 2. Different ratios of VPE and PE were blended in the torque rheometer at 130 °C for 10 min. The blends with different test requirements were prepared by a flat vulcanizing machine. The samples were irradiated with ^60^C_o_ γ-ray irradiation source with a dose of 300 kGy.

### 2.4. Fourier-Transform Infrared Spectroscopy (FTIR)

FTIR spectroscopy of 4′-hydroxyacetophenone and VPE were performed by a nicolet iS5 spectrometer (Madison, WI, USA) and spectra from 500 cm^−1^ to 4000 cm^−1^ were recorded. The resolution is 4 cm^−1^ with a total of 32 scans.

### 2.5. Nuclear Magnetic Resonance Hydrogen (^1^H NMR) spectroscopy

The ^1^H NMR spectra was carried out on a Bruker AVANCE III (Karlsruhe, Germany) at 400 MHz (^1^H) using CDCl_3_ as a solvent.

### 2.6. Samples Extraction

The samples were loaded in copper mesh bags and placed in the Soxhlet extractor (Taizhou, Jiangsu, China). Distilled water was added into a flat-bottomed flask, which was heated to boiling through a heating jacket. the samples were extracted for 24 h. after extraction, the samples were dried at 60 °C in a vacuum oven for 4 h.

### 2.7. Thermogravimetric Analysis (TGA)

TGA was performed using a TG209 F3 instrument (Bavaria, Germany) at a heating rate of 10 °C/min in the temperature range from 50 °C to 500 °C in a nitrogen gas purge.

### 2.8. AC Breakdown Strength

The voltage breakdown tester (LJC-50 kV, Beijing, China) is used to test the breakdown strength of samples. The boost rate is 0.5 kV/s.

### 2.9. Electrical Tree Test

The sample was immersed in silicone oil. Electrical tree initiation and propagation were observed by a CCD camera system (Suzhou, China), the electric tree experiment was carried out under the 15 kV AC voltage needle-plate electrode device.

### 2.10. γ-Ray Irradiation

The samples were placed in plastic bags and vacuumed, and were irradiated by the ^60^C_o_ γ-ray irradiation source with a dose of 300 kGy, the irradiation dose rate is 20 kGy/h.

### 2.11. Thermal Aging of the Samples

Samples were carried out at 90 °C for six days in a convection oven. The migration of voltage stabilizers was accelerated.

## 3. Results

### 3.1. VPE Structural Characterization

**Figure 1:**
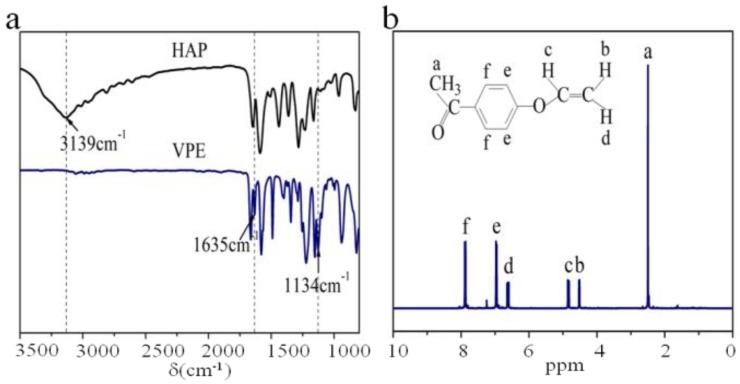
FTIR and ^1^H NMR analysis. (**a**) FTIR spectra of HAP and VPE; (**b**) ^1^H NMR spectra of VPE.

Fourier transform infrared (FTIR) spectrophotometry was conducted to investigate the structures of VPE, as shown in Figure 1a. The broad absorbance peak at 3139 cm^−1^ of 4′-hydroxyacetophenone is attributed to the stretching vibrations of phenolic hydroxyl groups exhibiting intermolecular hydrogen bonding [30]. The absorbance peak at 3139 cm^−1^ then disappears in VPE, while two new peaks appear at 1635 cm^−1^ and 1134 cm^−1^. The absorbance peak at 1134 cm^−1^ can be ascribed to the C–O–C stretching vibration, and the absorbance peak at 1635 cm^−1^ corresponds to the C=C stretching vibration. These results unambiguously demonstrate that a side group containing a double bond was introduced at the hydroxyl group.

Figure 1b shows the ^1^H NMR spectrum of VPE. The aromatic protons are observed at 7.04 and 7.93 ppm, while the C–CH_3_ group is observed at 2.57 ppm, the peak at 4.59, 4.93 and 6.69 ppm were assigned to the H of HC=CH_2_. The ratio of the integrated intensities of a, b, c, d, e, and f is 3:1:1:1:2:2, which matches well with the values calculated from the structures. The ^1^H NMR results confirm the successful preparation of VPE.

### 3.2. VPE Graft Characterization

**Figure 2:**
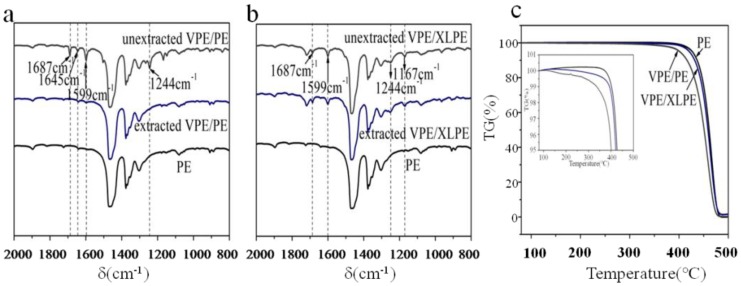
TGA and FTIR analysis. (**a**) infrared spectrum pre-and post-extraction of VPE/PE; (**b**) infrared spectrum pre-and post-extraction of graft VPE/XLPE; (**c**) TGA of PE, VPE/PE, graft VPE/XLPE.

FTIR was conducted to determine whether VPE was grafted onto the PE molecular chain and whether thermal migration of VPE was suppressed. The spectrum of uncrosslinked composite material exhibited characteristic absorption of C=C at 1645 cm^−1^, but this peak was not present in the spectrum of the crosslinked composite material, confirming that VPE was grafted onto XLPE. Figure 2a shows that the characteristic absorption peaks of the uncrosslinked composite material were significantly changed before and after extraction. The absorbance peaks of VPE at 1687 cm^−1^, 1645 cm^−1^, 1599 cm^−1^ and 1244 cm^−1^ correspond to C=O stretching vibrations, skeletal C=C in plane-stretching vibrations, an aromatic ketone absorption peak, and C–O–C stretching vibration which disappears after extraction, respectively. This indicates that VPE migrates during the extraction process. As shown in Figure 2b, the characteristic absorption peak of VPE still exists after the extraction of the crosslinked sample, indicating that the grafting reaction can effectively inhibit the thermal migration of VPE.

Thermal gravimetric analysis (TGA) curves of PE, VPE/PE, and graft VPE/XLPE are shown in Figure 2c. In the VPE/PE curve, the first stage of weight loss is the migration of VPE in the range of 120–225 °C. The weight loss of graft VPE/XLPE in the range of 120–225 °C is obviously less than that of VPE/PE, which indicates that graft VPE/XLPE can effectively inhibit VPE migration and enhance the thermal stability of the composite material.

### 3.3. Electrical Properties of Composite Materials

**Figure 3:**
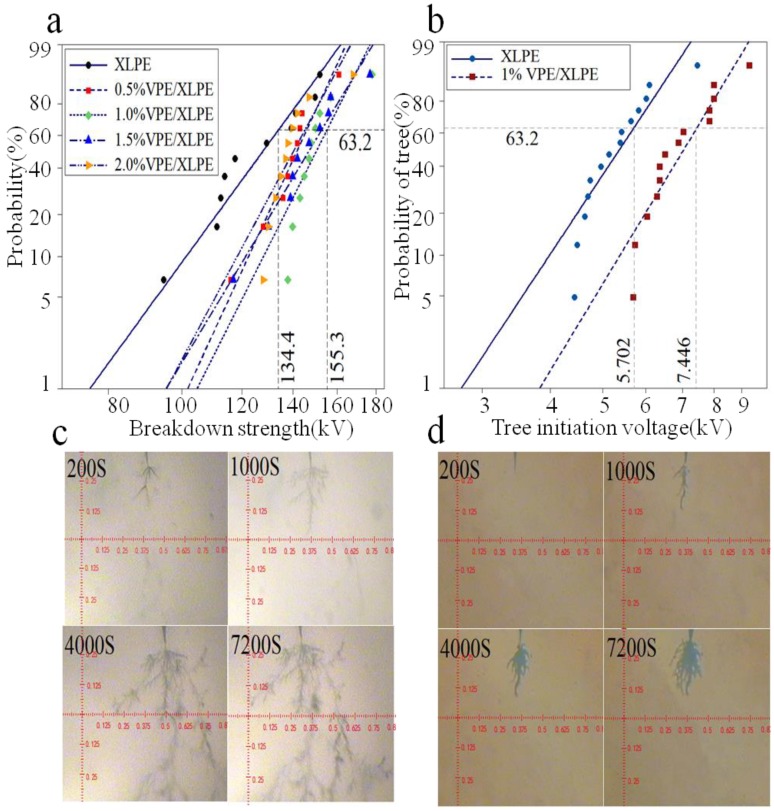
(**a**) breakdown strength Weibull curve of VPE/XLPE; (**b**) electrical tree initiation voltage of XLPE and composite; (**c**) electrical tree microscopic morphology of XLPE; (**d**) electrical tree microscopic morphology of VPE /XLPE.

Figure 3a shows the Weibull distribution of AC breakdown strength of VPE/XLPE composites with different proportions of VPE, and the shape parameter and breakdown strength are summarized in Table 1. The obtained results demonstrate that the addition of VPE can effectively improve the AC breakdown strength of composite materials. VPE can absorb the energy of high energy electrons through interactions and dissipate it through exothermic and other reactions, which prevents the high energy electrons from colliding with the polymer chains and breaking them, this improves electrical breakdown, which is a major cause of electrical tree mechanism in insulation materials [31]. The breakdown strength first increases and then decreases with the addition of VPE. At 1 wt %, the breakdown strength of the composite was increased the most, up to 15.5% compared to that of the pure XLPE.

Figure 3b–d show the inception, propagation, and shape features of the electrical tree in XLPE and VPE/XLPE. The electrical tree initiation voltages of XLPE and VPE/XLPE are shown in Figure 3b and summarized in Table 2. The results show that VPE can significantly improve the electrical tree initiation voltage of XLPE. The addition of 1 wt % of VPE increases the electrical tree initiation voltage by 30.6% as compared to pure XLPE. Figure 3c,d show the propagation and shape features of an electrical tree in XLPE and VPE/XLPE. The photos in Figure 3c,d show the propagation and shape features of an electrical tree in XLPE and VPE/XLPE. Electrical tree emerged in XLPE in 200 s, while there was no electrical tree in VPE/XLPE composite in 200 s, which indicated that VPE could inhibit the initiation of electrical tree. With increasing time, electrical tree in XLPE grew rapidly and formed branch-type. The growth rate of electrical tree in VPE/XLPE composite was significantly slower than that in XLPE. After 1000 s, the length of electrical tree in VPE/XLPE composite was almost unchanged, however, the width of electrical tree increased and presented a crowded bush-type structure. Thus, we can see from Figure 3 that VPE can enhance the breakdown strength and increase the threshold of electrical tree formation in the composite and significantly inhibit the initiation and propagation of electrical tree.

### 3.4. Thermal Aging

Heat aging can accelerate the migration of the voltage stabilizer. In order to investigate the influence of the migration of VPE in the XLPE composite on its dielectric properties, the AC breakdown strength and electrical tree initiation voltage of samples were measured before and after thermal aging and the results were compared.

**Figure 4:**
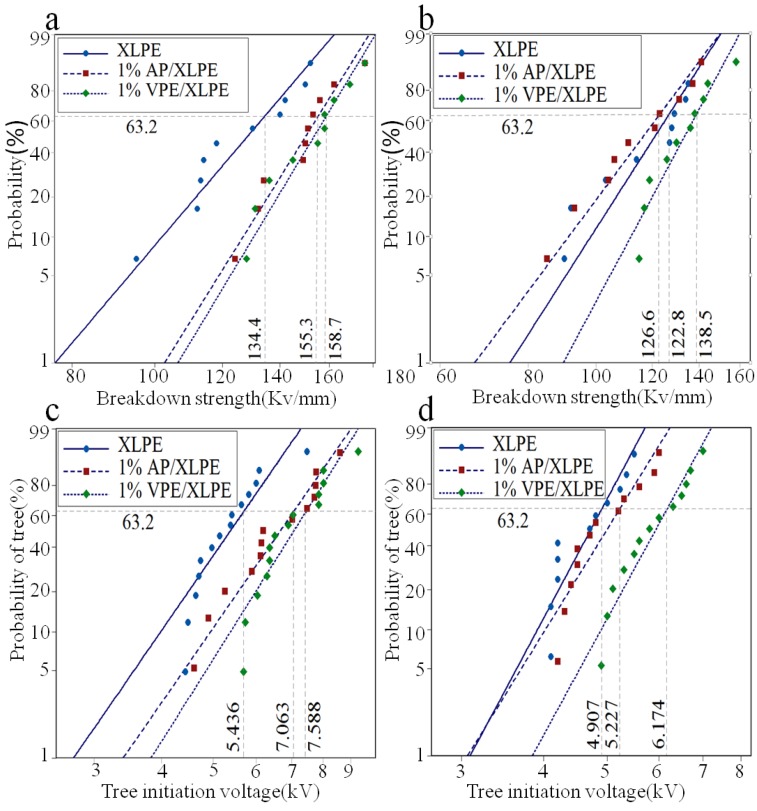
(**a**) breakdown strength of Thermal aging before; (**b**) breakdown strength of Thermal aging after; (**c**) electrical tree initiation voltage of Thermal aging before; (**d**) electrical tree initiation voltage of Thermal aging after.

The breakdown strength of the samples was tested before and after thermal aging. Weibull curves of breakdown strength are shown in Figure 4a,b, and the results are summarized in Table 3. From Figure 4a,b, we can see that the breakdown strength of the materials differed before and after heat aging. As can be seen from Figure 4a, VPE and AP can improve the breakdown strength of XLPE and have similar effects. Figure 4b shows that the breakdown strength decreased to some extent after heat aging. The breakdown strength of AP/XLPE decreased the most, with a breakdown strength of 155.3 kV/mm before heat aging and 122.8 kV/mm after heat aging, showing a decrease of 20.9%. The decrease in breakdown strength of VPE/XLPE was smaller than that of AP/XLPE. The electrical tree initiation voltage also shows the same trend before and after thermal aging. The electrical tree initiation voltages of XLPE, AP/XLPE and VPE/XLPE are shown in Figure 4c,d, and the electrical tree initiation voltages before and after thermal aging are summarized in Table 4. Figure 4c shows that acetophenone and VPE can significantly improve the electrical tree initiation voltage of XLPE before thermal aging, with increases of 29.9% and 39.6%, respectively. After thermal aging, as we can see from Figure 4d, the electrical tree initiation voltages of XLPE, AP/XLPE and VPE/XLPE decreased by 12.5%, 26.0% and 18.6%, respectively. The electrical tree initiation voltage of AP/XLPE was the most reduced, with that of VPE/XLPE being the second. This is mainly due to the poor compatibility between the voltage stabilizer and XLPE, with thermal aging accelerating the migration of voltage stabilizer in XLPE, which significantly reduced the breakdown strength and electrical tree initiation voltage of the materials. Because VPE was grafted onto the PE molecule chain during the radiation cross-linking process, migration of VPE was suppressed. However, as not all the VPE was grafted onto the PE molecule chain, some VPE moved from the bulk to the surface, resulting in a greater reduction in inception voltage for VPE/XLPE compared to that for XLPE during thermal aging.

## 4. Conclusions

We have described the synthesis and characterization of VPE, a grafted voltage stabilizer that can be grafted onto XLPE molecule chains during the crosslinking process. The addition of VPE can greatly improve the AC breakdown strength and electrical tree initiation voltage of the VPE/XLPE composite, and the inception and propagation of electrical tree were inhibited under HVAC conditions. Thermal aging experiments showed that VPE/XLPE insulation materials exhibit excellent permanent insulation performance, with superior results compared to AP/XLPE, which should be ascribed to the grafting of VPE onto XLPE, and the associated suppression of the migration of voltage stabilizers. This method is not only applicable to VPE/XLPE composites, but also to other aromatic voltage stabilizers and provides a way to improve the compatibility of aromatic voltage stabilizers with XLPE.

## Figures and Tables

**Table 1:** Weibull distribution parameters of AC breakdown strength.

Sample	Shape	AC Breakdown Strength (kV/mm)
XLPE	8.14	134.4
0.5% VPE/XLPE	12.78	146.0
1% VPE/XLPE	11.11	155.3
1.5% VPE/XLPE	9.79	152.8
2% VPE/XLPE	10.98	145.3

**Table 2:** Weibull distribution parameters of electrical tree initiation voltage.

Sample	Shape	Electrical Tree Initiation Voltage (kV)
XLPE	6.30	5.702
1% VPE/XLPE	6.92	7.446

**Table 3:** AC breakdown strength of different samples of Thermal aging before and after.

Sample	AC Breakdown Strength before Thermal Aging (kV)	AC Breakdown Strength After Thermal Aging (kV)	Heat Aging Loss Ratio (%)
XLPE	134.4	126.6	5.8
AP/XLPE	155.3	122.8	20.9
VPE/XLPE	158.7	138.5	12.7

**Table 4:** Electrical tree initiation voltage of different samples of Thermal aging before and after.

Sample	Tree Initiation Voltage before Thermal Aging (kV)	Tree Initiation Voltage after Thermal Aging (kV)	Heat Aging Loss Ratio (%)
XLPE	5.436	4.907	12.5
AP/XLPE	7.063	5.227	26.0
VPE/XLPE	7.588	6.174	18.6

## References

[1] Dissado L.A. (2002). Understanding electrical trees in solids: From experiment to theory. IEEE Trans. Dielectr. Electr. Insul..

[2] Bahadoorsingh S., Rowland S.M. (2010). Investigating the impact of harmonics on the breakdown of epoxy resin through electrical tree growth. IEEE Trans. Dielectr. Electr. Insul..

[3] Sarathi R., Nandini A., Tanaka T. (2012). Understanding electrical treeing phenomena in XLPE cable insulation under harmonic AC voltages adopting UHF technique. IEEE Trans. Dielectr. Electr. Insul..

[4] Du B.X., Ma Z.L., Gao Y., Han T. (2011). Effect of ambient temperature on electrical treeing characteristics in silicone rubber. IEEE Trans. Dielectr. Electr. Insul..

[5] Chen X.R., Mantsch A.R., Hu L.B., Gubanski S.M. (2014). Electrical treeing behavior of DC and thermally aged polyethylenes utilizing wire-plane electrode geometries. IEEE Trans. Dielectr. Electr. Insul..

[6] Liu Y., Cao X.L. (2015). Electrical tree growth characteristics in XLPE cable insulation under DC voltage conditions. IEEE Trans. Dielectr. Electr. Insul..

[7] Ashtiani M.B., Shahrtash S.M. (2013). On-line decision tree-based insulation assessment employing mathematical morphology filters for HV cables. IEEE Trans. Dielectr. Electr. Insul..

[8] Zheng X.Q., Chen G. (2008). Propagation mechanism of electrical tree in XLPE cable insulation by investigating a double electrical tree structure. IEEE Trans. Dielectr. Electr. Insul..

[9] Zhou K., Huang M., Tao W.B., He M. (2016). A possible water tree initiation mechanism for service-aged XLPE cables: Conversion of electrical tree to water tree. IEEE Trans. Dielectr. Electr. Insul..

[10] Yamano Y. (2014). Control of electrical tree at initiation stage in LDPE by mixed addition of Al_2_O_3_ nano-particle and azobenzoic compound. IEEE Trans. Dielectr. Electr. Insul..

[11] Bostrom J.O., Marsden E., Hampton R.N., Nilsson U. (2003). Electrical stress enhancement of contaminants in XLPE insulation used for power cables. IEEE Electr. Insul. Mag..

[12] Chen X., Murdany D., Liu D. (2016). AC and DC pre-stressed electrical trees in LDPE and its aluminum oxide nanocomposites. IEEE Trans. Dielectr. Electr. Insul..

[13] Wang S.J., Zha J.W., Li W.K., Dang Z.M. (2016). Distinctive electrical properties in sandwich-structured Al_2_O_3_/low density polyethylene nanocomposites. Appl. Phys. Lett..

[14] Li S., Min D., Wang W. (2016). Linking traps to dielectric breakdown through charge dynamics for polymer nanocomposites. IEEE Trans. Dielectr. Electr. Insul..

[15] Helal E., David E., Frechette M., Demarquette N.R. (2017). Thermoplastic elastomer nanocomposites with controlled nanoparticles dispersion for HV insulation systems: Correlation between rheological, thermal, electrical and dielectric properties. Eur. Polym. J..

[16] Liu D., Hoang A.T., Pourrahimi A.M. (2017). Influence of nanoparticle surface coating on electrical conductivity of LDPE/Al_2_O_3_ nanocomposites for HVDC cable insulations. IEEE Trans. Dielectr. Electr. Insul..

[17] Liu D., Pallon L.K.H., Pourrahimi A.M., Zhang P. (2016). Cavitation in strained polyethylene/aluminium oxide nanocomposites. Eur. Polym. J..

[18] Wutzel H., Jarvid M.J., Bjuggren M., Johansson A. (2015). Thioxanthone derivatives as stabilizers against electrical breakdown in cross-linked polyethylene for high voltage cable applications. Polym. Degrad. Stab..

[19] Zhang H., Shang Y., Zhao H., Han B.Z. (2015). Study of the effect of valence bond isomerizations on electrical breakdown by adding acetophenone to polyethylene as voltage stabilizers. Comput. Theor. Chem..

[20] Englund V., Huuva R., Gubanski S.M., Hjertberg T. (2009). High efficiency voltage stabilizers for XLPE cable insulation. Polym. Degrad. Stab..

[21] Yamano Y., Iizuka M. (2009). Suppression of Electrical Tree Initiation in LDPE by Additives of Polycyclic Compound. IEEE Trans. Dielectr. Electr. Insul..

[22] Jarvid M., Johansson A., Kroon R. (2014). A new application area for fullerenes: Voltage stabilizers for power cable insulation. Adv. Mater..

[23] Jarvid M., Johansson A., Englund V. (2015). High electron affinity: A guiding criterion for voltage stabilizer design. J. Mater. Chem. A.

[24] Zhang H., Shang Y., Zhao H., Han B.Z., Li Z.S. (2013). Mechanisms on electrical breakdown strength increment of polyethylene by acetophenone and its analogues addition: A theoretical study. J. Mol. Model..

[25] Englund V., Hjertberg T., Andersson M. (2013). Polyolefin composition for medium/high/extra high voltage cables comprising benzil-type voltage stabiliser. U.S. Patent.

[26] Jarvid M., Johansson A., Bjuggren J.M., Wutzel H. (2014). Tailored side-chain architecture of benzil voltage stabilizers for enhanced dielectric strength of cross-linked polyethylene. J. Polym. Sci. Part B.

[27] Englund V., Huuva R., Gubanski S., Hjertberg T. (2009). Synthesis and efficiency of voltage stabilizers for XLPE cable insulation. IEEE Trans. Dielectr. Electr. Insul..

[28] Zhang H., Shang Y., Li M.X., Zhao H. (2015). Theoretical study on the radical reaction mechanism in the cross-linking process of polyethylene. RSC Adv..

[29] Zhang H., Shang Y., Zhao H., Wang X. (2017). Theoretical study on the reaction of maleic anhydride in the UV radiation cross-linking process of polyethylene. Polymer.

[30] Gurnule W.B., Rahangdale P.K., Paliwal L.J., Kharat R.B. (2003). Synthesis, characterization and ion-exchange properties of 4-hydroxyacetophenone, biuret and formaldehyde terpolymer resins. React. Funct. Polym..

[31] Yamano Y. (2006). Roles of polycyclic compounds in increasing breakdown strength of LDPE film. IEEE Trans. Dielectr. Electr. Insul..

